# Examining the Genetic Background of Porcine Muscle Growth and Development Based on Transcriptome and miRNAome Data

**DOI:** 10.3390/ijms19041208

**Published:** 2018-04-16

**Authors:** Katarzyna Ropka-Molik, Klaudia Pawlina-Tyszko, Kacper Żukowski, Katarzyna Piórkowska, Grzegorz Żak, Artur Gurgul, Natalia Derebecka, Joanna Wesoły

**Affiliations:** 1 Department of Animal Molecular Biology, Laboratory of Genomics, National Research Institute of Animal Production, Krakowska 1, 32-083 Balice, Poland; klaudia.pawlina@izoo.krakow.pl (K.P.-T.); katarzyna.piorkowska@izoo.krakow.pl (K.P.); artur.gurgul@izoo.krakow.pl (A.G.); 2 Department of Cattle Breeding, National Research Institute of Animal Production, Krakowska 1, 32-083 Balice, Poland; kacper.zukowski@izoo.krakow.pl (K.Ż.); grzegorz.zak@izoo.krakow.pl (G.Ż.); 3 Laboratory of High Throughput Technologies, Institute of Molecular Biology and Biotechnology, Faculty of Biology, Adam Mickiewicz University, Umultowska 89, 61-614 Poznań, Poland; nataliad@amu.edu.pl (N.D.); j.wesoly@amu.edu.pl (J.W.)

**Keywords:** RNA-seq, miRNA, pig, next-generation sequencing (NGS), transcript analysis, muscle

## Abstract

Recently, selection in pigs has been focused on improving the lean meat content in carcasses; this focus has been most evident in breeds constituting a paternal component in breeding. Such sire-breeds are used to improve the meat quantity of cross-breed pig lines. However, even in one breed, a significant variation in the meatiness level can be observed. In the present study, the comprehensive analysis of genes and microRNA expression profiles in porcine muscle tissue was applied to identify the genetic background of meat content. The comparison was performed between whole gene expression and miRNA profiles of muscle tissue collected from two sire-line pig breeds (Pietrain, Hampshire). The RNA-seq approach allowed the identification of 627 and 416 differentially expressed genes (DEGs) between pig groups differing in terms of loin weight between Pietrain and Hampshire breeds, respectively. The comparison of miRNA profiles showed differential expression of 57 microRNAs for Hampshire and 34 miRNAs for Pietrain pigs. Next, 43 genes and 18 miRNAs were selected as differentially expressed in both breeds and potentially related to muscle development. According to Gene Ontology analysis, identified DEGs and microRNAs were involved in the regulation of the cell cycle, fatty acid biosynthesis and regulation of the actin cytoskeleton. The most deregulated pathways dependent on muscle mass were the Hippo signalling pathway connected with the TGF-β signalling pathway and controlling organ size via the regulation of ubiquitin-mediated proteolysis, cell proliferation and apoptosis. The identified target genes were also involved in pathways such as the FoxO signalling pathway, signalling pathways regulating pluripotency of stem cells and the PI3K-Akt signalling pathway. The obtained results indicate molecular mechanisms controlling porcine muscle growth and development. Identified genes (*SOX2*, *SIRT1*, *KLF4*, *PAX6* and genes belonging to the transforming growth factor beta superfamily) could be considered candidate genes for determining muscle mass in pigs.

## 1. Introduction

In pigs, as one of the major domesticated meat animals, processes regulating muscle growth and development have been widely investigated. However, despite numerous studies focused on the regulation of skeletal muscle growth and performed on various breeds, the obtained findings did not provide a clear view of the process. The next-generation sequencing (NGS) technology provides new possibilities for gene expression measurements and can be helpful to identify the genetic background of phenotypic traits [1].

In pigs, the analysis of global gene expression profiles using NGS technology in semimembranosus and longissimus dorsi muscles indicated genes potentially related to muscle growth [2,3]. Furthermore, the global gene expression profile was also analysed in terms of porcine meat quality [4]. Authors compared muscle transcriptomes of three pig breeds (Pietrain, Polish Landrace, and Pulawska pig) characterized by different production traits and selected genes potentially being under long-term selection focusing on improving the meat content in carcasses. Other studies confirmed the significant role of miRNAs in skeletal muscle growth and development as well as muscle disorders (atrophies and myopathies) [5,6,7]. The comparison of miRNA profiles between prenatal and postnatal ontogenesis periods allowed us to detect miRNAs and their muscle-specific targets associated with muscle development [8]. Tang et al. [9], performing a comprehensive analysis of miRNA-mRNA profiles in muscle tissue of local pig breeds, indicated the set of genes and miRNAs critical to prenatal skeletal muscle development.

A broad range of miRNA effects on gene expression and the translation process indicate the necessity of a comprehensive analysis of global gene and miRNA expression profiles. Such an approach will allow the detection of mechanisms responsible for gene expression regulation in the processes of muscle growth and development. The aim of the present research was to comprehensively identify genes and miRNAs potentially related to muscle growth in pig species. The RNA-seq method was applied to compare whole gene expression and miRNA profiles of muscle tissue collected from two sire-line pig breeds, which are characterized by high muscularity but differ in terms of this feature within each breed.

## 2. Results

### 2.1. Transcriptome Analysis—Differentially Expressed Genes (DEGs)

According to the RNA sequencing data, an average of 17.5 M reads per sample were obtained. On average, 98.3% of all sequences passed quality control (QC), of which 75% (12.6 M) mapped to the reference genome. Additionally, 74% of reads were mapped to exons, 18% to intronic regions and 8% to intergenic regions.

**Figure 1:**
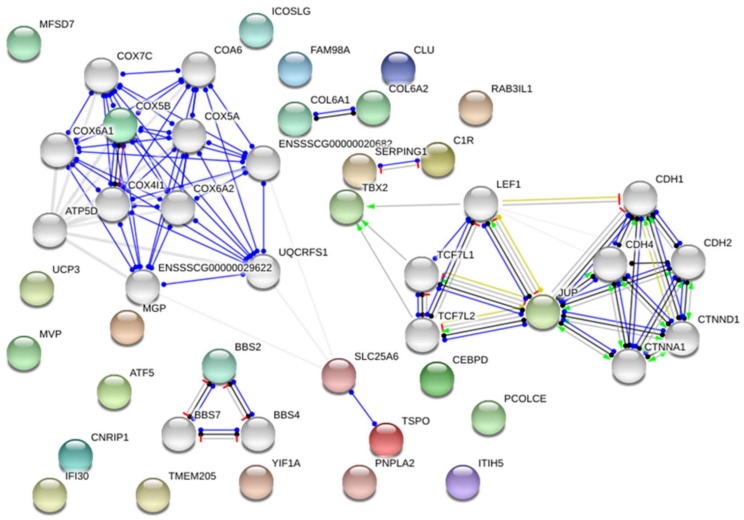
The differentially expressed genes were up-regulated in muscles of pigs with higher muscle mass. Detected genes were coloured and showed no more than 20 interactions (genes marked in grey); line shape indicates the predicted mode of action. Lines coloured indicate the predicted mode of action (red—interactions that were experimentally determined; blue—interactions from curated databases; black—co-expression; green—text mining associations and interactions based on relevant publications mentioning a transfer from other organisms, yellow—transcriptional regulation). Network were presented as a scalable vector graphic and based on *Sus scrofa* reference.

**Figure 2:**
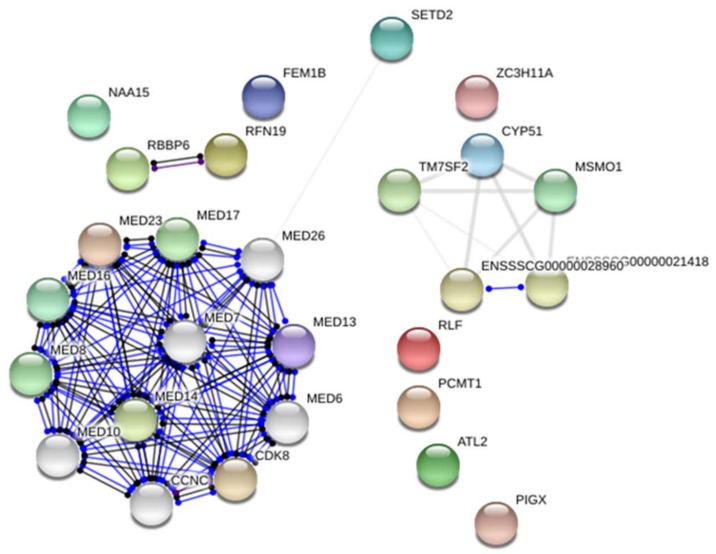
The differentially expressed genes were down-regulated in muscles of pigs with higher muscle mass. Detected genes were coloured and showed no more than 20 interactions (genes marked in grey); line shape indicates the predicted mode of action. Lines coloured indicate the predicted mode of action (blue—interactions from curated databases; black—co-expression). Network were presented as a scalable vector graphic and based on *Sus scrofa* reference.

The RNA-seq approach allowed the identification of 627 and 416 DEGs between the groups with different loin weights (adjusted *p*-value < 0.05; fold change ≤ 1.2) for Pietrain and Hampshire, respectively. Next, the comparison of obtained gene sets showed 43 DE genes identified in both breeds (Table S1). The up-regulated (30 genes) and down-regulated (13 genes) genes in muscles of pigs with higher muscle mass are presented in Figure 1 and Figure 2, respectively. Gene Ontology analysis showed that the detected genes were involved in the regulation of “metabolic process” (14 genes—*CYP51*, *ATGL*, *TBX2*, *CEBPD*, *RLF*, *JUP*, *RNF19A*); “cellular process” (GO:0009987) (10 genes—*RNF19A*, *UCP3*, *NAA15*, *ATGL*, *PCMT1*, *JUP*) and “developmental process” (GO:0032502) (3 genes—*CEBPD*, *TBX2*, *SETD2*). According to David software, several of the genes were classified as involved in “developmental growth” or “skeletal system development” (*PCOLCE*, *BBS2*, *TBX2*, *RBBP6*, *ATAF5*). The most over-represented DEGs encoded protein classes related to signal transduction, nucleic acid binding and enzyme modulators (Table 1).

In both breeds, in the pigs with higher muscularity, the most up-regulated *UCP3* gene coding for mitochondrial uncoupling protein 3 was primarily expressed in skeletal muscle and regulating the tissue respiratory processes (fold change 2.73 and 1.68 for Hampshire and Pietrain, respectively). On the other hand, genes showing the greatest significant expression decrease in the pigs with higher loin weight (compared to the animals with lower muscle mass) were *MED13* (encoding Mediator Complex Subunit 13; FC = −2.40 and −1.39 for Hp and Pi pigs) and *CYP51* (encoding cytochrome P450 family 51 subfamily A member 1; FC = −2.26 and −1.41 for Hp and Pi pigs) genes.

The RNA-seq experiment was submitted to the NCBI Gene Expression Omnibus (GEO) functional genomics data and assigned the GEO accession number GSE107207.

### 2.2. miRNAome Analysis—Differentially Expressed miRNA

As a result of the miRNAome sequencing, between 2.5 M and 7.6 M raw sequences in individual samples were identified. After the filtering, between 2.4 M (94.6%) and 5.8 M (77%) sequences were further mapped to the reference genome.

**Figure 3:**
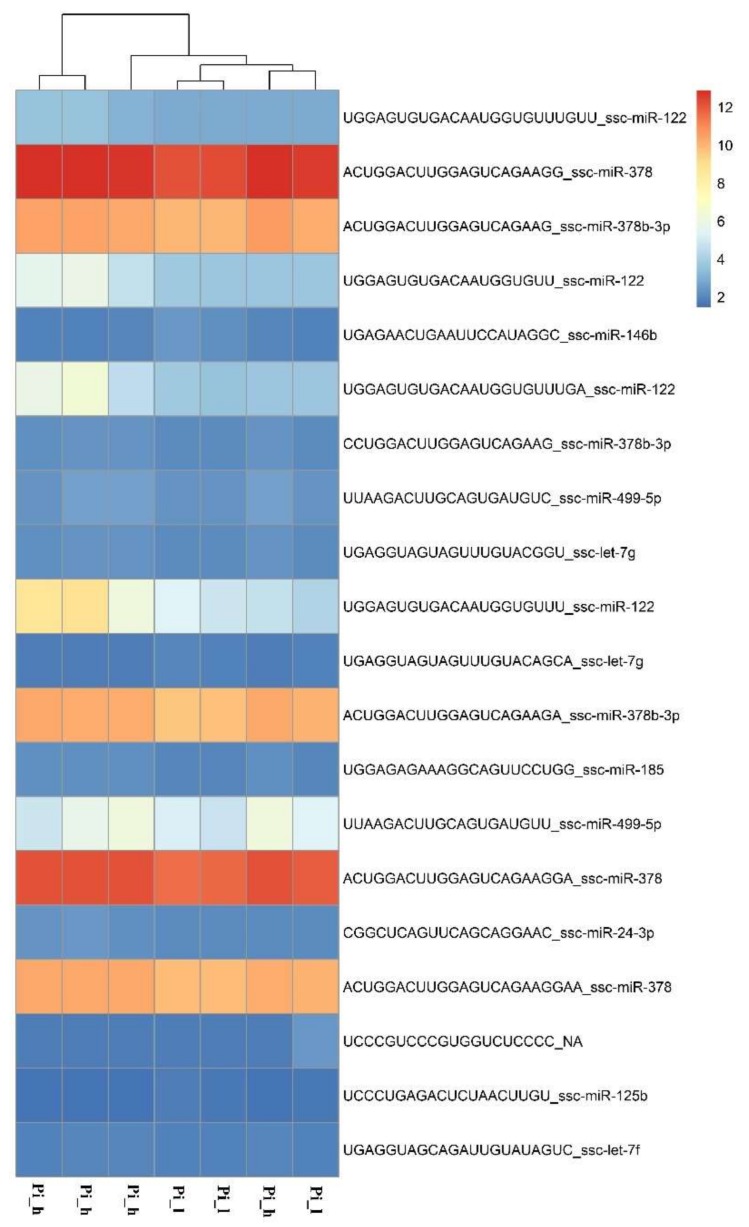
The expression pattern obtained for the most significant (*p* < 0.01) miRNAs detected in Pietrain breed in pig groups differed in muscle weight (h, l,—high or low muscle mass) (R Package pheatmap, R Foundation for Statistical Computing, Vienna, Austria).

**Figure 4:**
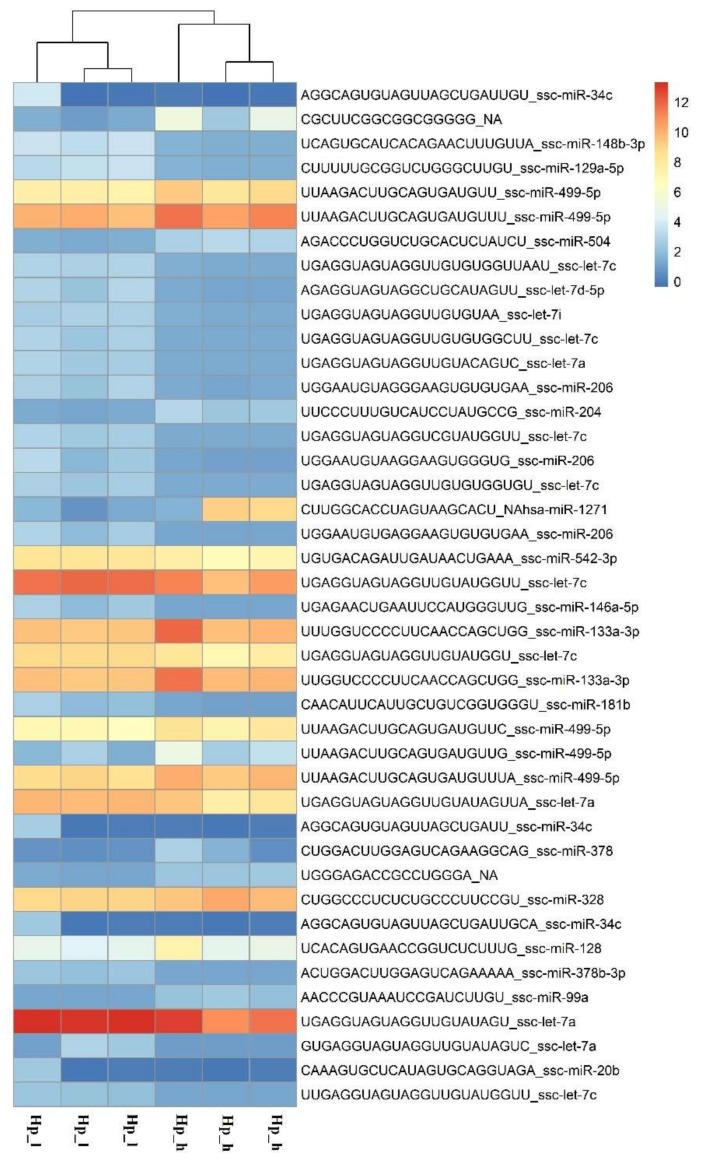
The expression pattern obtained for the most significant (*p* < 0.01) miRNAs detected in the Hampshire breed in pig groups differed in muscle weight (h, l,—high or low muscle mass) (R Package pheatmap).

miRNAome sequencing allowed the detection of 196 known and 149 potentially new microRNAs in both breeds. In the Pietrain samples, 160 known and 59 potentially novel miRNAs were identified, while for the Hampshire pigs, 192 known and 138 potentially new miRNAs were detected. The comparison of miRNA profiles between pig groups with different loin weights showed differential expression of 57 microRNAs for Hampshire pigs (24 up-regulated and 34 down-regulated; Figures S1 and S2) and 34 miRNAs for Pietrain pigs (28 up-regulated and 6 down-regulated; Figures S3 and S4). The most significant (*p* < 0.01) miRNAs detected in Pietrain and Hampshire pigs are shown in Figure 3 and Figure 4. Furthermore, 18 common microRNAs with differential expression were identified for both tested breeds (miR-499a-5p; miR-206; miR-146a-5p; miR-133a-3p; miR-378b; miR-128-3p; miR-378b-3p; miR-10b; miR-451a; miR-125b; miR-30a-5p; let-7f-5p; let-7i-5p; let-7g-5p; miR-7-5p; miR-26a-5p; miR-185-5p; miR-126-3p). This finding may indicate the existence of muscle growth with the regulating pathways common for both investigated high-muscularity pig breeds.

**Figure 5:**
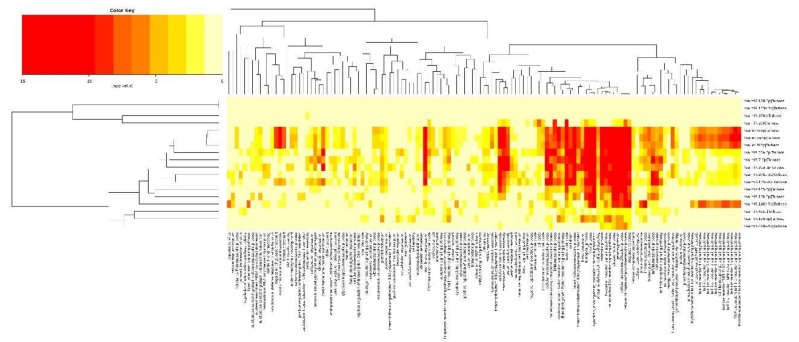
The identified Gene Ontology biological processes related to 18 microRNAs commonly detected in both breeds as differentially expressed between pigs with various muscle masses (DIANA-miRPath v3.0, DIANA-Lab, Athens, Greece).

Eighteen DE microRNAs detected in both breeds were further subjected to Gene Ontology and KEGG Pathway analyses (mirPath v.3.0, DIANA-Lab, Athens, Greece). According to an experimentally validated target gene database (Tarbase v7.0, (DIANA-Lab, Athens, Greece)), the most over-represented GO processes were identified, such as cell cycle, fatty acid biosynthesis, regulation of actin cytoskeleton as well as lysine degradation and ubiquitin mediated proteolysis (Table 2). All identified GO biological processes related to 18 DE microRNAs are shown in Figure 5. The GO terms in each breed and taking into account the direction of expression change is presented in Figures S1–S4.

The miRNA experiment was submitted to the NCBI GEO database and assigned the GEO accession number GSE109215.

### 2.3. Integrated Analysis of Transcriptome and miRNAome

**Figure 6:**
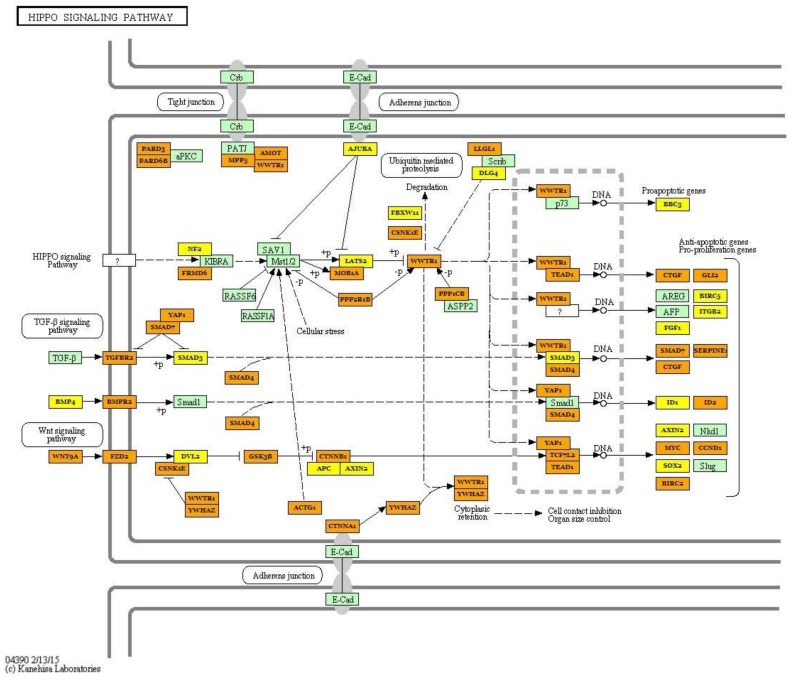
The Hippo signalling pathway identified as the most regulated by detected differentially expressed miRNAs depends on muscle mass. Green frame represents genes engaged in the pathway. Targeted genes regulated by identified miRNAs were marked orange (genes included in more than one pathway) and yellow (genes included only in one pathway) (KEGG database; hsa04390). Black arrows stand for a molecular interaction or relation, while dotted arrows stand for an indirect link or unknown reaction.

**Figure 7:**
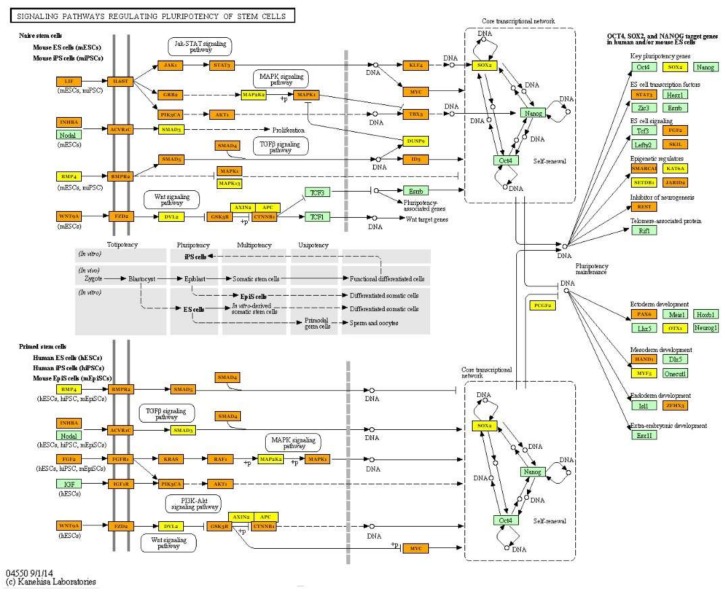
The signalling pathways regulating pluripotency of stem cells regulated by detected differentially expressed miRNAs depend on muscle mass. Green frame represents genes engaged in the pathway. Targeted genes regulated by identified miRNAs were marked orange (genes included in more than one pathway) and yellow (genes included only in one pathway) (KEGG database; hsa04550). Black arrows stand for a molecular interaction or relation, while dotted arrows stand for an indirect link or unknown reaction.

The pathway analysis based on 43 DEGs allowed us to identify three pathways in which two genes coding for collagens (*COL6A1* and *COL6A2*) were involved: the PI3K-Akt signalling (ssc04151), Focal adhesion (ssc04510) and ECM-receptor interaction (ssc04512) pathways. Furthermore, two genes (*CYP51*—Cytochrome P450 51A; *PIGX*—Phosphatidylinositol Glycan Anchor Biosynthesis Class X) belonging to the metabolic pathway (ssc01100) were identified. The KEGG pathway analysis based on miRNA results indicated that the most significant pathway was the Hippo signalling pathway, which is also connected with the TGF-β signalling pathway and controls organ size via the regulation of ubiquitin-mediated proteolysis, cell proliferation and apoptosis (Figure 6). The most interesting target genes involved in identified pathways (FoxO signalling pathway, TGF-β signalling pathway, signalling pathways regulating pluripotency of stem cells and PI3K-Akt signalling pathway) were *SOX2*, *SIRT1*, *KLF4*, *PAX6* (Figure 7) and other genes belonging to the transforming growth factor beta superfamily (Table 3).

Moreover, the analysis of miRNA–target gene interactions was performed with the use of the DIANA-mirExTra v2.0 [10] (DIANA-Lab, Athens, Greece) tool to identify functional microRNAs potentially responsible for changes in gene expression identified in the present study as DEGs. mRNA and microRNA differential expression analysis results were simultaneously analysed, and important miRNA regulators were identified based on functional analysis of their target genes. The obtained results confirmed the significant association of 5 detected microRNAs and 9 DEGs (Table 4). Two genes were identified as the most frequently regulated by detected miRNAs, namely, *CYP51A1*, which is related to cholesterol metabolism (hsa-miR-155-5p; hsa-miR-30c-5p; hsa-miR-199b-5p), as well as the *RNF19A* gene, which is associated with ubiquitination (hsa-miR-155-5p; hsa-miR-133a-3p; hsa-miR-126-5p) (Table 4).

### 2.4. Validation of the Obtained Results

The estimation of gene expression using real-time PCR confirmed the direction of transcript level changes between analysed biological groups detected using the RNA-seq approach (FC profiles values). The Pearson correlation calculated between relative quantity (RQ) values and normalized read counts (RNA-seq) showed high significant correlation coefficients (from 0.643 to 0.928) for most of the analysed genes (Table 5). For the *TBX2* and *XPO1* genes, the correlation was not significant, but the FC values obtained by these two methods were similar. The validation of miRNA showed a high and significant correlation between NGS and qPCR results for most of the analysed miRNAs (from 0.635 to 0.888) (Table 6). For two miRNAs, miR-7-5p and miR-125b-5p, the obtained correlation coefficients were not significant (0.402 and 0.232, respectively).

## 3. Discussion

Since the beginning of domestication, selection in pigs has been focused on improving the lean meat content in carcasses. Currently, this direction of selection is most evident in breeds constituting a paternal component in breeding (Duroc, Pietrain and Hampshire pigs), which are used to improve the meat quantity of cross-breed pig lines. Furthermore, even in one breed, a significant variation in the meatiness level can be observed. In the present study, a comprehensive analysis of genes and microRNA profiles in porcine muscle tissue was performed to identify the genetic background of meat content in carcasses. Moreover, NGS analyses were applied to compare global genes and miRNA expression profiles between groups differing in weight of loin muscle in each breed. Such an approach allowed us to avoid a potential breed-specific effect on transcript abundance and to detect differential expression of genes related to the trait of interest.

The global gene expression profile in a cell or tissue is a result of the transcription rate as well as other post-transcriptional modifications, including mRNA silencing. One of the main gene’s expression regulators is microRNA molecules, which predominantly promote transcript degradation [11]. Commonly, miRNAs regulate transcript stability via binding to the 3’UTR of their targeted genes with the RISC multiprotein complex (RNA-induced silencing complex) [12]. microRNAs can control transcript silencing by involvement in transcriptional inhibition through microRNA-mediated chromatin reorganization, mRNA destabilization by decay or cleavage and regulation of proteins belonging to transcription complexes [13,14].

To date, efforts have been made to identify regulatory mechanisms related to muscle growth and development during ontogenesis in pigs. Using a pathway-focused oligo microarray, Li et al. [15] compared the expression profile of genes involved in muscle growth and fatty acid biosynthesis in muscle tissue collected from two pig breeds at six growth stages. Among the genes significantly deregulated during growth periods, the authors detected the *UCP3* gene (uncoupling protein 3), whose expression was regulated by 30 other identified genes. The *UCP3* gene encodes mitochondrial anion carrier protein, and together with *UCP2*, it is essential for body metabolism regulation [16]. Both genes are mainly expressed in muscle tissue and control mitochondrial fatty acid transport and glucose metabolism [17]. In our research, we confirmed the differential expression of the *UCP3* gene between pigs with varied muscling in both breeds. Furthermore, a miRNA–mRNA target analysis showed an interaction between the *UCP3* gene and identified in Hampshire pigs miR-30c-5p, which was established to regulate myoblast differentiation [18]. MicroRNAs belonging to the miR-30 family also affect the activity of miR-206—the main modulator of skeletal muscle development [19,20]. Muscle-specific miR-206 (myomiR) was detected in the present study as differentially expressed in Pietrain and Hampshire pigs differing in muscle weight. This observation confirmed that miR-206 plays an important role in muscle growth and development, regardless of breed tested.

Sheng et al. [21] performed whole miRNAome profiling in pig breeds with high lean meat percentage (Large White) and low body weight (Min Pigs) at several postnatal stages and showed that the one of the most abundant myomiRs was miR-206. Interesting results were reported by Cai et al. [22], who confirmed that the microRNA profile in skeletal muscle was significantly altered by castration. The authors identified seven differentially expressed miRNAs between intact and castrated pigs: miR-206, let-7c and let-7a (down-regulated) and miR-1, miR-133a, miR-26a, and miR-133b (up-regulated). Cai et al. [22] indicated the key impact of castration on miRNA expression, which can explain the main mechanism underlying variations in muscle growth in intact and castrated pigs. Our study allowed us to detect three of seven previously described miRNAs (miR-206; miR-26a; miR-133a). Moreover, we detected the expression of three microRNAs (let7i; let-7g; let-7f) belonging to the let-7 family, which are also known as important developmental regulators.

Previous studies performed by Huang et al. [23] and Yan et al. [24] showed that skeletal muscle hypertrophy can be controlled by the IGF-1–Akt pathway and myostatin signalling pathway. It results from the regulation of *IGF-1* or *IGFR* expression by miR-133a/b and miR-206. Furthermore, during exercise-induced muscle response, IGF-1 signalling can be modified by miR-126, which also impacts *MyoD* and *Myf5* genes strongly related to myogenesis [25]. In humans, several muscle-specific miRNAs, including miR-133a/b and miR-206, are associated with muscular dystrophies (Duchenne and Becker) and are called dystromirs. Their serum expression profile can be used as a biomarker of such muscle disorder [26]. Apart from miR-206 and miR-133a, our results showed significant differences in miR-126-3p expression and significant changes in the expression of its target gene, *RNF19A* (Ring Finger Protein 19A). It has been established that the *RNF19A* gene mainly plays a role in neurons causing amyotrophic lateral sclerosis or Parkinson’s disease. On the other hand, it was proposed as one of interesting genes related to muscle response during stabilized weight loss [27].

Another gene with modified transcript levels dependent on loin mass was the *SETD2* gene, whose expression is regulated by miR-30c-5p. The *SETD2* gene, which encodes a histone methylotransferase, is involved in the regulation of transcription processes via histone methylation. Recent studies have shown that the *SETD2* gene is involved in epigenetic mechanisms regulating myoblast proliferation and differentiation [28]. Furthermore, using the CRISPR/CAS9 system (Clustered Regularly-Interspaced Short Palindromic Repeats), the authors indicated that silencing the *SETD2* gene resulted in a decrease in the expression of major regulators of the cell cycle, repression of myogenin—MyoG—transcription and up-regulation of the cyclin-dependent kinase inhibitor p21. The arrest of the cell cycle caused by the silencing of the *SETD2* gene demonstrated that this gene is critical during myoblast proliferation and differentiation [28]. In our study, a significant up-regulation of the *SETD2* gene in pigs characterized by higher muscle mass in carcasses was observed. These results support previous findings and indicate a strong relationship between the *SETD2* gene and myogenesis in pigs.

The comparison of the global miRNA profile in muscle tissue in the pigs differing in terms of muscularity allowed for the detection of some pathways regulated by identified differentially expressed miRNAs. The most significantly enriched pathways were the Hippo signalling pathway, FoxO signalling pathway and signalling pathways regulating pluripotency of stem cells. Numerous studies have shown that the Hippo signalling pathway plays a key role in muscle cell proliferation, differentiation and apoptosis and, as a result, controls myogenesis processes [24,29,30,31]. The Hippo signal transduction network can regulate muscle mass and organ size via the activation of cascades of serine/threonine kinases [32,33]. Moreover, Gnimassou et al. [33] suggested that this pathway can contribute to changes and adaptation of muscle mass after exercise. microRNAs identified in the present study can be upstream regulators of the Hippo pathway and, as described previously, are considered to be myomiRs closely related to muscle growth. Our results showed that several miRNAs (miR-30a-5p; miR-206; miR-26a-5p; miR-499a-5p; miR-146a-5p) also regulated the FoxO, TGF-β, PI3K-Akt signalling pathways and signalling pathways regulating pluripotency of stem cells. The identified miRNAs regulate genes coding for proteins belonging to the 14-3-3 family (*YWHAE*; *YWHAB*; *YWHAQ*), which are considered important versatile regulators of the cell cycle, metabolism, cell signalling and apoptosis [34,35]. It was established that 14-3-3 proteins act through regulation of FoxO transcription factors—especially FoXO3, whose significant role in controlling modifications of muscle mass (hypertrophy or atrophy) was confirmed [36]. Furthermore, the identified microRNAs control the transcript abundance of genes regulating different aspects of development: TGF-β proteins (*TGFBR1*; *BMP2*; *BMP4BMP5*; *BMPR1B*; *BMPR2*) and genes recognized as pluripotency markers (*SOX2*; *KLF4).* The deregulation of the detected DE miRNAs and genes can provide new information about processes related to the growth and development of muscle tissue in pigs.

The *PCOLCE* gene (Procollagen C-Endopeptidase Enhancer), which is involved in muscle growth disorders in humans, was detected in the set of DEGs in Pietrain and Hampshire breeds. This gene encodes an enzyme responsible for the transformation of procollagens to collagens and is associated with the deregulation of extracellular matrix (ECM) functions and, as a result, with the appearance of late-onset muscle fibrosis and oculopharyngeal muscular [37]. The extracellular matrix plays a key role during signal transduction, and any disorders of this function can lead to myopathies. However, the exact mechanism of the association between impaired ECM matrix function and muscle growth remains unknown. The differential expression of the *PCOLCE* gene in muscle tissue diverting in mass suggests that this gene can contribute to porcine muscle growth via modification of the ECM role. In both breeds, the differential expression of two genes coding for collagens were detected, namely, *COL6A1* and *COL6A2.* Both *COL6A2* and its paralog *COL6A1* contribute to the organization of matrix components by binding several extracellular matrix proteins and are related to the occurrence of muscle dystrophy and myopathy (bethlem mopathy 1 and ullrich congenital muscular dystrophy 1) [38,39]. These findings also support the hypothesis that ECM matrix remodelling is associated with the variation of muscle tissue growth and development.

Furthermore, the *TBX2* gene coding for the T-box transcription factor was identified in our study as up-regulated in pigs with higher muscle mass. In humans, it was confirmed that *TBX2* is responsible for developing tissue sarcomas in which this gene is overexpressed. Zhu et al. [40] indicated that *TBX2* regulates the skeletal muscle cell cycle via an interaction with the myogenic transcription factor MyoD and the cell cycle regulators p21 and p14. The authors suggested that in sarcomas, *TBX2* promoted cell proliferation, and inhibition of its expression led to the repression of tumour growth [40,41].

## 4. Materials and Methods

### 4.1. Animals

RNA-seq and miRNAome analyses were performed on *longissimus lumborum* samples collected from 13 pigs representing two sire-line breeds—Pietrain and Hampshire. Animals were maintained at the same housing and feeding conditions according to the procedure previously described [42]. When animals reached a weight of 105 kg (±2.5), they were slaughtered, and after 24 h of chilling, the half carcasses were dissected. Based on dissection data, pigs were selected from a larger population in terms of weight of loin muscle to obtain groups with possibly extreme phenotypes in each breed (Table 7). Pigs included in the present study were unrelated (minimally three crosses back).

The research was performed on biological material derived from pigs maintained and slaughtered in the Test Pig Station (National Research Institute of Animal Production), where pigs are slaughtered, dissected and after carcass evaluation, meat is standard intended for consumption. Therefore, research performed does not require the approval of Animal Experimentation committee.

Immediately after slaughter, the tissue samples were collected into the RNAlater solution (Ambion, Thermo Fisher Scientific, Waltham, MA, USA) and stored at −20 °C. Total RNA was isolated from muscle tissue using Direct-zol RNA Mini Prep (Zymo Research, Irvine, CA, USA). The quality and quantity of the obtained RNA were checked using a NanoDrop 2000 spectrophotometer (Thermo Fisher Scientific; Waltham, MA, USA) and a TapeStation 2200 system (RNAScreen Tape, Agilent Technologies, Santa Clara, CA, USA). Samples with RIN (RNA integrity number) values above 7.5 were used to construct libraries.

### 4.2. Transcriptome Sequencing and Data Analysis

The cDNA libraries were constructed from 300 ng of total RNA with the TruSeq RNA Sample Prep Kit v2 kit (Illumina, San Diego, CA, USA) according to the protocol. The quantification of obtained libraries was performed with Qubit 2.0 Fluorometer (Invitrogen, Thermo Fisher Scientific, Waltham, MA, USA) and TapeStation 2200 instrument (D1000 ScreenTape; Agilent Technologies, Santa Clara, CA, USA). The mRNA sequencing was performed in 101 single-end run on a HiScanSQ platform (Illumina) with the use of TruSeq SR Cluster Kit v3—CBOT-HS and TruSeq SBS Kit v3–HS (Illumina). The libraries were indexed with different adaptors, pooled and sequenced in 4 technical replicates.

The pipeline used for raw sequence data quality control (QC), estimation of gene transcript levels and differential expression analysis were described in detail previously [43]. The obtained raw reads were aligned to the *Sus scrofa* reference genome (assembly Sscrofa10.2 (GCF_000003025.5)). Differentially expressed genes (DEGs; fold change ≥ |1.2|; adjusted *p*-value  <  0.05) were determined separately for each breed using Deseq2 software ( version 1.12.4, [44] between pig groups differing in weight of loin muscle. The GO and pathway analyses were performed using Panther and David software (version 13.1 and v6.8; respectively) [45,46] with *Sus scrofa* reference. The *p*-value in enrichment tests was determined based on the Mann–Whitney U Test (Wilcoxon Rank-Sum Test) (Panther software, v 1.12.4 [45], while significantly enriched pathways were detected based on Fisher’s exact test (David software, v6.8) [46].

### 4.3. miRNAome Sequencing and Data Analysis

MicroRNA libraries were prepared using the NEBNext Multiplex Small RNA Library Prep Set for Illumina (New England Biolabs, Ipswich, MA, United States) according to the standard protocol. Briefly, the first step was the 3′ adaptor ligation, followed by hybridization with the Reverse Transcription Primer and ligation with the 5′ adaptor. The RNA-adaptor ligation products were subjected to reverse transcription. Then, PCR amplification with 12 different indexed primers was performed to allow further multiplexing of the samples. The amplified samples were purified and size-selected on a Novex 6% TBE PAGE gel (Invitrogen, Thermo Fisher Scientific, Waltham, MA USA). After the overnight elution from the gel, the libraries were precipitated and purified with ethanol. Next, they were subjected to a concentration measurement with a Qubit 2.0 Fluorometer (Thermo Fisher Scientific) and a size assessment with a 2200 TapeStation instrument (Agilent). Finally, the obtained libraries were pooled and sequenced by applying 36 cycles on HiScan SQ (Illumina) according to the manufacturer’s protocol.

The obtained reads were converted to FastQ files, subjected to demultiplexing using bcl2fastq software (version, Illumina), and quality controlled using FastQC software [47]. The obtained sequences were analysed using UEA sRNA Workbench v3.2 [48]. The first step was 3′ adaptor trimming and filtering according to the following parameters: minimum abundance of at least 6 supporting reads, 17–25 nt of length, low complexity as well as tRNA and rRNA sequences removed. MicroRNAs were identified by applying the miRCat tool v1.0with default parameters for animals, except for minimum abundance (6 reads), minimum length (17 nt), and maximum length (25 nt). The *Sus scrofa* genome (assembly Sscrofa 10.2, Ensemble database) and miRBase v21.0 (Griffiths-Jones lab at the Faculty of Biolo Manchester; USA) [49,50] were used as references. Detected potentially novel miRNAs were additionally controlled for the presence of other non-coding RNAs using the RNAcentral database (The RNAcentral Consortium) [51]. In the last step, the identification of microRNA length and sequence variants—isomiRs—was performed. The analysis was carried out using isomiR-SEA software (Version 1.60) [52] with the default settings.

Differential expression analysis using DESeq2 software (v.1.12.4) [44] was carried out for each breed separately, and significantly expressed miRNAs (*p* ≤ 0.05) were chosen for further analyses.

miRNAs identified as differentially expressed in the examined samples were analysed using the mirPath v.3.0 DIANA Tools web application (DIANA-Lab, Athens, Greece) [53] to identify their target genes and biological processes enriched by them. DIANA—TarBase v7.0 (DIANA-Lab, Athens, Greece) [54] with experimentally validated target genes was used as a reference database of target genes, and the KEGG Pathway Database was used as a reference database of biological pathways. The analysis was performed on the basis of human homologues deposited in miRBase (21.0) due to the lack of data for pig microRNAs.

### 4.4. Validation of NGS Data by qPCR

The validation of RNA-seq results was performed for 6 genes. The exact transcript abundance was measured using real-time PCR analysis. The primers for all genes were designed using Primer 4.0 software based on the Ensemble reference genome. cDNA was synthesized from 200 ng of total RNA using a High-Capacity RNA-to-cDNA™ Kit (Applied Biosystems, Thermo Fisher, Waltham, MA, USA) according to the protocol. qPCR was performed in 45 cycles on a QuantStudio 7Flex system (Applied Biosystems) with three replicates for each sample (AmpliQ 5x HOT EvaGreen qPCR Mix; Novazym; Poland). *OAZ1* and *RPL27* were used as endogenous controls. The details of the validated genes are presented in Supplementary File 1—Table. The expression levels were calculated using the delta-delta Ct method [55]. The correlation coefficients between RQ (relative quantity) values and normalized read counts (RNA-seq) were calculated using Pearson’s correlation analysis (SAS software, v. 8.02, Wadowice, Poland)).

RT-qPCR method was also employed to validate the expression levels of 9 selected microRNAs in all samples (Table S2). Reverse transcription reactions were performed using a TaqMan Advanced miRNA cDNA Synthesis Kit (Thermo Fisher Scientific) according to the manufacturer’s protocol. Next, quantitative PCR reactions were run using TaqMan Fast Advanced Master Mix (Thermo Fisher Scientific) and commercially available TaqMan microRNA Advanced Assays (Thermo Fisher Scientific), following the protocol and including Non-Template Control (NTC) for each microRNA assay. The reactions were run in triplicate on a QuantStudio 7 Flex Real-Time PCR System (Thermo Fisher Scientific). The efficiency of amplification reactions for each of the miRNA assays was calculated based on the standard curve method.

The NormFinder software [56] was employed to choose an endogenous control that is a microRNA with the most stable expression profile (miR-103a-3p; miR-20a-5p). Finally, relative expression levels of the examined microRNAs were calculated using the ΔΔCt method, including reaction efficiency E [55].

## 5. Conclusions

The comprehensive analysis of the transcriptome and miRNAome profile in porcine muscle tissue differing in terms of muscle mass allowed us to identify differentially expressed genes and miRNAs potentially related to muscle growth and development. We detected microRNAs characteristic of muscle tissue, such as microRNAs belonging to the miR-30 family, miR-206, miR-26a and miR-133a, which have been established as the main modulators of skeletal muscle development. The identification of DE genes and microRNAs enabled us to pinpoint interesting molecular pathways and biological processes essential in controlling muscle growth and development in the pig. The close relationship of some identified in the present study genes and miRNAs with muscle disorders in humans can confirm their significant role in the myogenesis process in pigs. The presented molecular networks show new possibilities for searching candidate genes related to the process of developing muscle tissue and as a result with important production traits in pigs.

## Figures and Tables

**Table 1:** The detected significant Gene Ontology terms based on genes differentially expressed between pigs differing in terms of muscle mass.

GO Term	*p-*Value/Genes	Number of Genes Detected/Genes		Hp	Pi
Signal transduction	0.034	11		FC	FC
	ENSSSCG00000013901	*IFI30*	IFI30, lysosomal thiol reductase	1.82	1.37
	ENSSSCG00000009668	*CLU*	clusterin	1.92	1.45
	ENSSSCG00000028022	*COL6A2*	collagen type VI alpha 2 chain	1.71	1.36
	ENSSSCG00000022236	*FOLR1*	folate receptor 1 (adult)	2.01	1.53
	ENSSSCG00000022246	*ICOSLG*	inducible T-cell costimulator ligand	1.83	1.39
	ENSSSCG00000011129	*ITIH5*	inter-alpha-trypsin inhibitor heavy chain family member 5	1.67	1.38
	ENSSSCG00000000606	*MGP*	matrix Gla protein	2.04	1.30
	ENSSSCG00000026934	*PIGX*	phosphatidylinositol glycan anchor biosynthesis class X	1.27	1.36
	ENSSSCG00000027466	*PCOLCE*	procollagen C-endopeptidase enhancer	2.23	1.27
	ENSSSCG00000013181	*SERPING1*	serpin family G member 1	2.38	1.27
	ENSSSCG00000014834	*UCP3*	uncoupling protein 3	2.73	1.68
Nucleid acid binding	0.010	9			
	ENSSSCG00000014834	*UCP3*	uncoupling protein 3	2.73	1.68
	ENSSSCG00000007830	*RBBP6*	RB binding protein 6, ubiquitin ligase	2.06	1.25
	ENSSSCG00000027060	*TBX2*	T-box 2	1.73	1.39
	ENSSSCG00000011332	*SETD2*	SET domain containing 2	1.87	1.27
	ENSSSCG00000006276	*CEBPD*	CCAAT/enhancer binding protein delta	2.57	1.38
	ENSSSCG00000027946	*MVP*	major vault protein	1.32	1.53
	ENSSSCG00000003670	*RLF*	rearranged L-myc fusion	1.80	1.25
	ENSSSCG00000026631	*SLC25A6*	solute carrier family 25 Member 6	1.73	1.35
	ENSSSCG00000028035	*ZC3H11A*	zinc finger CCCH-Type Containing 11A	2.73	1.68
Cytoplasm	0.032	5			
	ENSSSCG00000025417	*BBS2*	Bardet-Biedl syndrome 2	1.37	1.35
	ENSSSCG00000009668	*CLU*	clusterin	1.92	1.45
	ENSSSCG00000017428	*JUP*	junction plakoglobin	1.31	1.39
	ENSSSCG00000004103	*PCMT1*	protein-L-isoaspartate (D-aspartate) O-methyltransferase	1.46	1.26
	ENSSSCG00000006066	*RNF19A*	ring finger protein 19A, RBR E3 ubiquitin protein ligase	2.10	1.31
Enzyme modulator	0.015	4			
	ENSSSCG00000003201	*ATF5*	activating transcription factor 5	1.51	1.54
	ENSSSCG00000013181	*SERPING1*	serpin family G member 1	2.38	1.27
	ENSSSCG00000011129	*ITIH5*	inter-Alpha (Globulin) Inhibitor H5	1.67	1.38
	ENSSSCG00000013074	*RAB3IL1*	RAB3A Interacting Protein Like 1	2.16	1.59
Cytoskeleton	0,021	3			
	ENSSSCG00000025417	*BBS2*	Bardet-Biedl syndrome 2	1.37	1.35
	ENSSSCG00000017428	*JUP*	junction plakoglobin	1.31	1.39
	ENSSSCG00000006066	*RNF19A*	ring finger protein 19A, RBR E3 ubiquitin protein ligase	2.10	1.31
Methyltransferase	0.0066	3			
	ENSSSCG00000011332	*SETD2*	SET domain containing 2	1.87	1.27
	ENSSSCG00000015311	*CYP51*	cytochrome P450, family 51, subfamily A, polypeptide 1	2.27	1.41
	ENSSSCG00000004103	*PCMT1*	protein-L-isoaspartate (D-aspartate) O-methyltransferase	1.46	1.26

Hp—Hampshire; Pi—Pietrain; FC—fold change obtained for DEGs between different phenotypic groups in each breed.

**Table 2:** The detected significant Gene Ontology terms based on miRNAs differentially expressed between pigs differing in terms of muscle mass.

GO	Detected miRNA	N	Most Interesting Genes Related with GO Term	*p*-Value
**Cell cycle (hsa04110)**				1.026 × 10^−9^
	hsa-let-7i-5p	32	*CDK4*; *CDK2*; *CDK6*; *TP53; RBL2; ESPL1; ORC2*	
	hsa-let-7g-5p	33	*CDK4; CDK2; CDK6; TP53; RBL2; ESPL1; ORC2*	
	hsa-miR-30a-5p	24	*PCNA; SMAD2*; *TP53*; *MYC*; *YWHAE*	
	hsa-let-7f-5p	35	*ESPL1*; *CCNB2*; *RBL2*; *SMC1A*; *CDK4*; *TP53*; *ATR*	
	hsa-miR-499a-5p	7	*YWHAG*; *E2F5*; *SKP2*; *MYC*; *CDC27*; *PRKDC*; *MDM2*	
	hsa-miR-146a-5p	12	*GSK3B*; *CCNB1*; *CCNA2*; *SMAD4*; *RBL1*; *CDC23*; *PRKDC*; *MDM2*; *ABL1*	
	hsa-miR-378a-3p	20	*CCNB1*; *YWHAG*; *CCND2*; *MCM4*; *CDK1*; *BUB3*; *MDM2*	
	hsa-miR-10b-5p	13	*YWHALE*; *CDK2*; *CCND2*; *CUL1*; *TP53*; *CDC27*	
	hsa-miR-7-5p	20	*YWHAH*; *YWHAE*; *CCNB1*; *E2F2*; *CDK2*; *ATM*;	
	hsa-miR-26a-5p	32	*CDC6*; *CCNB1*; *YWHAE*; *YWHAG*; *YWHAZ*; *SMAD4*; *WEE1*; *EP300*	
	hsa-miR-185-5p	16	*CDK4*; *YWHAE*; *YWHAG*; *YWHAB*; *YWHAQ*; *ORC4*;	
	hsa-miR-126-3p	4	*E2F1*; *E2F3*; *CCNE2*; *CCNE1*	
	hsa-miR-125b-5p	19	*CDC6*; *E2F2*; *E2F3*; *ATR*; *TP53*; *CUL1*; *ORC5*	
	hsa-miR-206	9	*CDK4*; *CCND2*; *CDC7*; *WEE1*; *MCM7*; *CDC25C*	
**Fatty acid biosynthesis (hsa00061)**				
	hsa-miR-30a-5p	4	*FASN; ACSL1; ACSL3; ACSL4*	6.038 × 10^−9^
	hsa-miR-26a-5p	4	*FASN; ACSL1; ACSL3; ACSL4*	
	hsa-miR-378a-3p	2	*ACSL4; ACACA*	
	hsa-miR-7-5p	4	*FASN; OXSM; ACACA; ACSL4*	
	hsa-let-7i-5p	1	*MCAT*	
	hsa-let-7g-5p	1	*MCAT*	
	hsa-let-7f-5p	1	*MCAT*	
	hsa-miR-125b-5p	1	*FASN*	
**Regulation of actin cytoskeleto n (hsa04810)**				0.0218
	hsa-let-7i-5p	31	*PPP1CA; PFN1; BRAF; ACTB; SSH2; CRK; GNA13; FN1; RAC1; VAV3; MAPK1; FGFR1*	
	hsa-let-7g-5p	31	*PPP1CA; PFN1; BRAF; ACTB; SSH2; CRK; GNA13; FN1; RAC1; VAV3; MAPK1; FGFR1*	
	hsa-miR-125b-5p	27	*SSH2; EZR; NRAS; CRKL; CRK; PAK2; PXN; MYL9; FGFR2; FGFR1; MYH9*	
	hsa-let-7f-5p	32	*PPP1CA; PFN1; BRAF; ACTB; SSH2; CRK; GNA13; FN1; RAC1; VAV3; MAPK1; FGFR1*	
	hsa-miR-378a-3p	17	*ACTB; SSH2; ITGA9; CRKL; GNA13; PFN2; PAK4; MAPK1; MYLK*	
	hsa-miR-146a-5p	9	*BRK1; ROCK1; WASL; EDFR; ACTN1; ITGB2; WASF2*	
	hsa-miR-7-5p	35	*ACTB; ACTN2; NRAS; APC; RRAS2; FN1; FGF1; ACTN4; MAPK1; PIK3CB; EGFR*	
	hsa-miR-185-5p	14	*PIP5K1C; FN1; MYLK; ENAH; CDC42; ITGB5; RHOA; EGFR; PPP1CC*	
	hsa-miR-378b	4	*SSH2; ITGB1; ROCK2; MSN*	
	hsa-miR-26a-5p	32	*SSH2; EZR; NRAS; CRK; ITGA8; PAK1; ITGB3; ITGA6*	
	hsa-miR-30a-5p	29	*ARPC5; WASL; NRAS; SSH1; GNA13; EGFR; FN1; RAC1; PIK3CA; PPP1CB*	
	hsa-miR-10b-5p	6	*CRK; ARPC5; ACTG1; TIAMI; PIK3CD; DIAPH2*	
	hsa-miR-126-3p	8	*PIK3CA; PIK3CD; ITGA6; MAPK1; KRAS; CRK; PIK3R2*	
	hsa-miR-451a	3	*BRAF; WASL; PIK3CA*	
	hsa-miR-206	2	*EGFR; FN1*	
	hsa-miR-499a-5p	2	*CFL2; FGF2*	
**Lysine degradation (hsa00310)**				
	hsa-let-7i-5p	12	*WHC1L1; SETD1B; PLOD2; ASHIL; SETD1A; KMT2B*	7.854 × 10^−7^
	hsa-let-7g-5p	11	*PLOD2; SETD1B; ASH1L; DOT1L; KMT2D; KMT2E*	
	hsa-miR-125b-5p	7	*KMT2D; WHSC1; KMT2C; KMT2B; SUV39H1; SUV420H2*	
	hsa-let-7f-5p	12	*ALDH7A1; SETD1B; KMT2D; DOT1L; SETD1A; KMT2B; KMT2E*	
	hsa-miR-7-5p	15	*ALDH3A2; SETD1B; PLOD2; PLOD1; SETD2; NSD1; OGDH;*	
	hsa-miR-378a-3p	4	*SETD7; ASH1L; KMT2D; SUV420H1*	
	hsa-miR-30a-5p	12	*SETD7; PLOD2; OGDH; NSD1; SETD2; ALDH2*	
	hsa-miR-185-5p	3	*ASHIL; KMT2D; WHSC1*	
	hsa-miR-10b-5p	4	*SETD7; SUV420H1; WHSC1L1; ALDH3A2*	
	hsa-miR-26a-5p	9	*SETD2; NSD1; ACAT2; SETD8; SETDB1; KMT2D; KMT2C; KMT2A; ALDH9A1*	
	hsa-miR-146a-5p	2	*NSD1; KMT2C*	
**Ubiquitin mediated proteolysis (hsa04120)**				
	hsa-let-7i-5p	29	*BCBL; MAP3KI; UBE3B; BTRC; HUWEI; SKP2; NEDD4L; MID1U; BOX5*	9.766 × 10^−5^
	hsa-let-7g-5p	30	*UBE3B; CBL; CUL2; NEDD4L; MAP3K1; MID1; BIRC3; UBOX5; SKP2*	
	hsa-miR-30a-5p	42	*UBE3B; CUL2; NEDD4L; MAP3K1; MID1; BIRC3; UBOX5; SKP2; UBA6; SEA1*	
	hsa-let-7f-5p	29	*UBE2Z; CBL; UBE2R2; UBR5; STUB1; XIAP; FBXW7; BIRC6*	
	hsa-miR-7-5p	23	*UBE2Z; CBL; UBE2R2; UBR5; STUB1; XIAP; FBXW7; BIRC6*	
	hsa-miR-26a-5p	23	*WWP2; SMURF2; HUWE1; SKP1; BRCA1; CDC23; MDM2;*	
	hsa-miR-378a-3p	9	*UBE2Z; CBL; BTRC; FBXW7; HUWE1; PML; RBX1; MDM2*	
	hsa-miR-206	1	*BRCA1*	
	hsa-miR-146a-5p	6	*ITCH; MID1; BRCA1; TRAF6; CDC23; MDM2*	
	hsa-miR-10b-5p	11	*UBE2Z; UBE3B; CUL1; BRCA1; NEDD4; CDC27; MDM2*	
	hsa-miR-185-5p	12	*UBE2Z; UBR5; HUWE1; BIRC6; MDM2; TRIP12; UBE4A*	
	hsa-miR-125b-5p	10	*HUWE1; CUL1; UBA6; BIRC6; SAE1; CDC27; XIAP*	
	hsa-miR-499a-5p	4	*SKP2; CDC27; MDM2; KLHL9*	
	hsa-miR-126-3p	2	*UBE2K; FBXW11*	
	hsa-miR-378b	1	*RBX1*	

**Table 3:** The pathways and genes regulated by identified miRNAs (*N*-number of genes regulated in each pathway).

Pathway	miRNA Involved in Pathway Regulation	*n*	Target Genes	*p*-Value
Hippo signaling pathway (hsa04390)	hsa-miR-499a-5p; hsa-miR-30a-5p, hsa-miR-7-5p; hsa-let-7i-5p; hsa-let-7g-5p; hsa-miR-125b-5p; hsa-let-7f-5p; hsa-miR-26a-5p; hsa-miR-185-5p; hsa-miR-146a-5p;hsa-miR-10b-5p; hsa-miR-378a-3p; hsa-miR-451a; hsa-miR-126-3p; hsa-miR-206; hsa-miR-378b	87	*PPP1CA*; *ACTB*; *TGFBR1*; *BMP5*; *BMPR1B*; *WNT5A*; *YWHAE*; *WNT10B*; *WNT3A*; *WNT8B*; *BMP2*; *YWHAB*; *YWHAQ*; *MOB1B*; *SMAD4*; *BMP4*; *SOX2*; *BMPR2*	6.038 × 10^−9^
FoxO signaling pathway (hsa04068)	hsa-let-7i-5p; hsa-let-7g-5p; hsa-let-7f-5p; hsa-miR-206; hsa-miR-378a-3p; hsa-miR-30a-5p; hsa-miR-7-5p; hsa-miR-185-5p; hsa-miR-26a-5p; hsa-miR-126-3p; hsa-miR-499a-5p; hsa-miR-146a-5p; hsa-miR-451a; hsa-miR-125b-5p; hsa-miR-10b-5p	88	*BRAF*; *NRAS*; *KRAS*; *TGFBR1*; *TGFBR2*; *PIK3R2*; *FBX032*; *IGFR*; *SIRT1*; *IRS2*; *FOXO6*; *FOXO3*; *USP7*	2.159 × 10^−8^
TGF-β signaling pathway (hsa04350)	hsa-miR-10b-5p; hsa-let-7i-5p; hsa-let-7g-5p; hsa-miR-30a-5p; hsa-let-7f-5p; hsa-miR-26a-5p;hsa-miR-378a-3p; hsa-miR-499a-5p; hsa-miR-451a; hsa-miR-7-5p; hsa-miR-126-3p, hsa-miR-146a-5p; hsa-miR-125b-5p; hsa-miR-185-5p; hsa-miR-378b; hsa-miR-206	50	*FST*; *TGFBR1*; *ID2*; *ID4*; *ROCK1*; *SMAD2*; *SMAD3*; *SMAD4*; *SMAD7*; *SP1*; *BMP4*; *BMP2*; *BMP5*; *BMPR1B*; *BMPR2*; *MAPK1*	1.357 × 10^−6^
PI3K-Akt signaling pathway (hsa04151)	hsa-miR-30a-5p; hsa-miR-7-5p; hsa-let-7i-5p; hsa-miR-10b-5p; hsa-let-7g-5p; hsa-let-7f-5p; hsa-miR-125b-5p;hsa-miR-185-5p; hsa-miR-206; hsa-miR-378a-3p; hsa-miR-26a-5p; hsa-miR-499a-5p; hsa-miR-146a-5p; hsa-miR-126-3p;hsa-miR-451a;hsa-miR-378b	167	*TLR2*; *PRLR*; *MET*; *ITGB1*; *ATF2*; *CDK2*; *GNG5*; *GNG11*; *GNG12*; *CDK2*; *STK11*; *CK6*; *IGF1R*; *EGFR*; *TP53*; *PIK3CD*; *PIK3R3*; *FGF9*; *ITGA5*; *FGF1*	0.016
Signaling pathways regulating pluripotency of stem cells (hsa04550)	hsa-miR-125b-5p; hsa-miR-30a-5p; hsa-miR-378a-3p; hsa-miR-10b-5p; hsa-let-7i-5p; hsa-let-7g-5p; hsa-let-7f-5p; hsa-miR-7-5p; hsa-miR-26a-5p; hsa-miR-146a-5p; hsa-miR-185-5p; hsa-miR-126-3p; hsa-miR-499a-5p; hsa-miR-451a	86	*SOX2*; *KLF4*; *PAX6*; *PIK3R2*; *FZD3*; *MAPK13*; *KRAS*; *SMAD6*; *NRAS*; *IGF1R*; *TBX3*; *LIF*; *MYC*; *BPO2*; *FGF2*; *LIFR*; *CTNNB1*; *PIK3CA*; *SMP4*; *BMPR2*; *JAKI*	1.27 × 10^−6^

**Table 4:** The miRNA-mRNA interactions identified between differentially expressed miRNAs and genes based on the DIANA-miRextra v2.0 tool (DIANA-Lab, Athens, Greece).

Hampshire		Pietrain	
miRNA	*DEGs*	miRNA	*DEGs*
hsa-miR-155-5p	*RNF19A*; *FEM1B*; *ATL2*; *CYP51A1*; *XPO1*	hsa-miR-199b-5p	*CYP51A1*
hsa-miR-30c-5p	*SETD2*; *ATL2*; *PIGX*; *RBB6P*; *CYP51A1*; *XPO1*	hsa-miR-126-5p	*RNF19A1*
hsa-miR-133a-3p	*XPO1*; *RNF19A*; *PNPLA2*		

**Table 5:** The validation results obtained between the correlation of RNA-seq and qPCR data for differentially expressed genes.

Gene	Accession Number	Hampshire	Pietrain			*R*
		FC RNA-seq	FC RQ	FC RNA-seq	FC RQ	
*MGP*	ENSSSCG00000000606	2.45	2.07	1.63	1.80	0.928 ***
*BBS2*	ENSSSCG00000025417	−2.25	−1.76	−1.53	−1.03	0.646 ***
*PCOLCE*	ENSSSCG00000027466	3.35	2.79	1.57	1.10	0.886 ***
*COL6A1*	ENSSSCG00000022506	2.20	1.40	1.97	1.70	0.643 **
*TBX2*	ENSSSCG00000027060	2.05	1.91	2.21	1.09	0.360 ^ns^
*XPO1*	ENSSSCG00000028228	−1.71	−0.62	−1.50	−1.80	0.280 ^ns^

*R*—correlation coefficient; FC—fold change values between groups with high and low meat content in carcasses in each breed; RQ—relative quantity, Pearson correlation coefficient with *p*-value (***—*p* < 0.000; **—*p* < 0.01; ns—not significant).

**Table 6:** The correlation coefficient of RNA-seq and qPCR data obtained for miRNAs.

miRNA	*R*
let-7g-5p	0.738 **
miR-126-3p	0.794 **
miR-181b-5p	0.658 *
miR-30a-5p	0.888 ***
miR-378a-3p	0.635 *
miR-451a	0.756 **
miR-499a-5p	0.878 ***
miR-7-5p	0.402 ^ns^
miR-125b-5p	0.232 ^ns^

*R*—correlation coefficient; Pearson correlation coefficient with *p*-value (***—*p* < 0.0001; **—*p* < 0.01; *—*p* < 0.05; ns—not significant).

**Table 7:** The differences in muscle weight observed between the analysed groups for both breeds.

	Hampshire						Pietrain					
	Low (*n* = 3)			High (*n* = 3)			Low (*n* = 3)			High (*n* = 3)		
Weight of loin (kg)	54.0	±2.80	**b**	63.4	±2.05	**a**	57.9	±1.27	**B**	64.3	±0.41	**A**
Lean meat percentage	63.3	±1.73		66.1	±0.35		68.1	±2.45		69.8	±2.5	
Weight of ham (kg)	8.69	±0.31	**b**	10.37	±0.25	**a**	9.51	±0.54		9.83	±0.49	

Data are presented as means ± standard error; means with different letters differ significantly **a**,**b**—*p*-value ≤ 0.05; **A**,**B**—*p*-value ≤ 0.01).

## Supplementary Materials

Supplementary materials can be found at http://www.mdpi.com/1422-0067/19/4/1208/s1.
