# Supplementary material for: Examining the Genetic Background of Porcine Muscle Growth and Development Based on Transcriptome and miRNAome Data
Source: Int J Mol Sci. 2018 Apr 16;19(4):1208. doi: 10.3390/ijms19041208 (PMC5979540; doi:10.3390/ijms19041208)
Supplement: Supplementary file 1 [file ijms-19-01208-s001.pdf]

Supplementary Figures captions:

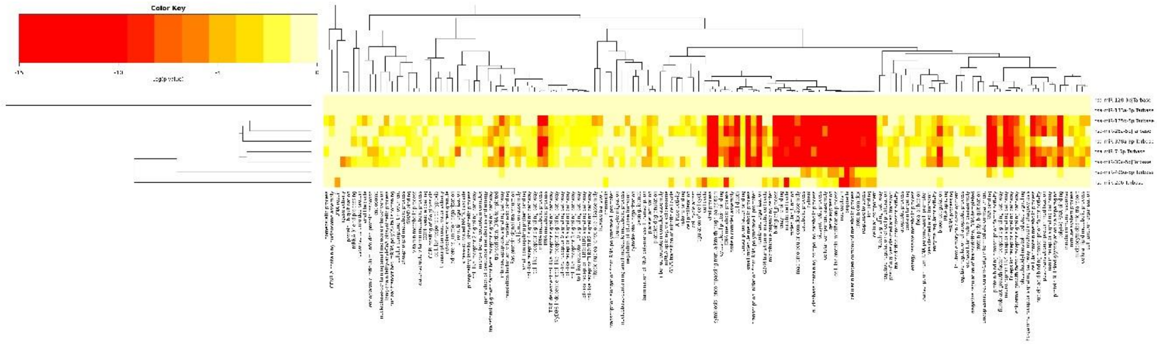

Figure S1. The identified Gene Ontology biological processes related to miRNAs up-regulated in pigs with higher muscle mass in the Hampshire breed (DIANA-miRPath v3. 0).

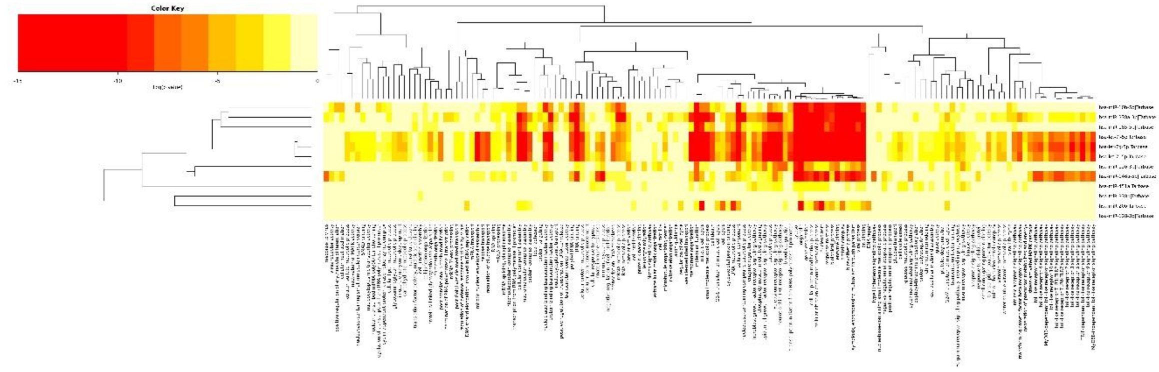

Figure S2. The identified Gene Ontology biological processes related to miRNAs up-regulated in pigs with higher muscle mass in the Hampshire breed (DIANA-miRPath v3. 0).

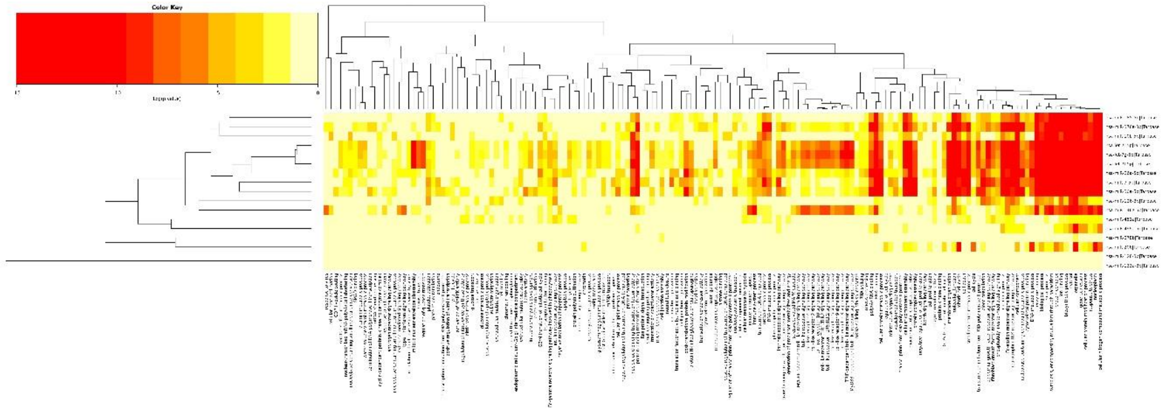

Figure S3. The identified Gene Ontology biological processes related to miRNAs up-regulated in pigs with higher muscle mass in Piertrain breed (DIANA-miRPath v3. 0).

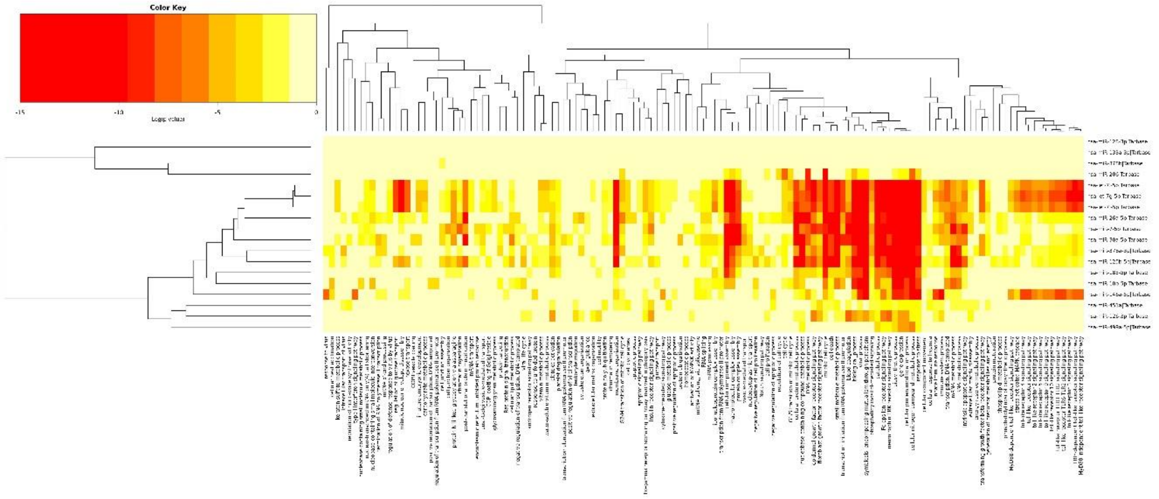

Figure S4. The identified Gene Ontology biological processes related to miRNAs down-regulated in pigs with higher muscle mass in Piertrain breed (DIANA-miRPath v3. 0).

Table S2. The details of differentially expressed genes and miRNAs selected to validation of NGS results.

**Genes**				
**Gene symbol**	**Gene name**	**Accession number**	**Primers**	**Amplicon lenght (bp)**
MGP	Matrix Gla Protein; Cell Growth-Inhibiting Gene 36 Protein	ENSSSCG00000000606	F AGACCCTGAGAGCAACCTCA<br>R GGAGGCTTGTTGAGTTCTCG	224
BBS2	Biedl-Bardet- Syndrome 2	ENSSSCG00000025417	F TGTGGCTCAACCAGAACTTT<br>R GGAAATCCGCTTCTACCTGA	225
PCOLCE	Procollagen C-Endopeptidase Enhancer	ENSSSCG00000027466	F CCTCTCCACCCACTGACACT<br>R TGGATAAGAAAGGGGTGCCTA	261
COL6A1	Collagen Type VI Alpha 1 Chain	ENSSSCG00000022506	F GCAACCTGGTGTGGAACG<br>R CTTTGATGCCCAGGTGCTT	312
TBX2	T-Box 2	ENSSSCG00000027060	F AGATCGACAACAACCCGTTT<br>R CAGGGGACTAGGCGCTGT	209

XPO1	Exportin 1	ENSSSCG00000028228	F TCACAATCAAGTGAATGGTACAGA R TTCCAGTGAGCTCTTAAAAATCG	241
**miRNAs**				
**miRBase ID**	**miRBase ID (Sus Scrofa)**	**miRBase Accession #**	**TaqMan Assay ID**	
hsa-miR-126-3p	ssc-miR-126-3p	MIMAT0018378 (pig)	477887_mir	
hsa-miR-378a-3p	ssc-miR-378	MIMAT0013868 (pig)	478349_mir	
hsa-let-7g-5p	ssc-let-7g	MIMAT0013867 (pig)	478580_mir	
hsa-miR-30a-5p	ssc-miR-30a-5p	MIMAT0010193 (pig)	479448_mir	
hsa-miR-451a	ssc-miR-451	MIMAT0018382 (pig)	478107_mir	
hsa-miR-125b-5p	ssc-miR-125b	MIMAT0002120 (pig)	477885_mir	
hsa-miR-7-5p	ssc-miR-7	MIMAT0000252 (human)	478341_mir	
hsa-miR-499a-5p	ssc-miR-499-5p	MIMAT0013877 (pig)	478139_mir	
hsa-miR-103a-3p	ssc-miR-103	MIMAT0002154 (pig)	478253_mir	
hsa-miR-20a-5p	ssc-miR-20a	MIMAT0000075 (human)	478586_mir	

Table S1. The 43 DEGs identified in both breeds between groups differing in meat content.

Gene Accession number	Gene symbol	Hp FC	adj pval	Pi FC	adj pval
ENSSSCG00000017674	*MED13*	-2.39	5.94E-11	-1.39	0.002140
ENSSSCG00000015311	*CYP51*	-2.27	5.24E-08	-1.41	0.007487
ENSSSCG00000006066	*RNF19A*	-2.10	1.84E-10	-1.31	0.006446

ENSSSCG00000007830	*RBBP6*	-2.06	6.78E-12	-1.25	0.033282
ENSSSCG00000028035	*ZC3H11A*	-2.04	5.09E-12	-1.24	0.038055
ENSSSCG00000009061	*NAA15*	-2.03	3.68E-12	-1.23	0.025751
ENSSSCG00000028228	*protein_coding*	-1.93	4.62E-06	-1.30	0.014457
ENSSSCG00000011332	*SETD2*	-1.87	3.00E-09	-1.27	0.016705
ENSSSCG00000003670	*RLF*	-1.80	3.23E-06	-1.25	0.045695
ENSSSCG00000022419	*FEM1B*	-1.74	2.50E-05	-1.25	0.031716
ENSSSCG00000008487	*ATL2*	-1.72	7.57E-06	-1.34	0.013031
ENSSSCG00000004103	*PCMT1*	-1.46	0.002628	-1.26	0.039723
ENSSSCG00000026934	*PIGX*	-1.27	0.24488	-1.36	0.021497
ENSSSCG00000026182	*protein_coding*	1.14	0.646066	1.54	0.003136
ENSSSCG00000014565	*protein_coding*	1.16	0.582601	1.34	0.048292
ENSSSCG00000029147	*protein_coding*	1.29	0.356686	1.41	9.87E-07
ENSSSCG00000017428	*JUP*	1.31	0.134578	1.39	0.025119
ENSSSCG00000027946	*MVP*	1.32	0.033742	1.53	0.002281
ENSSSCG00000025417	*BBS2*	1.37	0.127142	1.35	0.022786
ENSSSCG00000008349	*CNRIP1*	1.42	0.020235	1.42	0.001871
ENSSSCG00000022506	*COL6A1*	1.46	0.17294	1.37	0.032944
ENSSSCG00000003201	*ATF5*	1.51	0.041036	1.54	0.003205
ENSSSCG00000011129	*ITIH5*	1.67	0.000236	1.38	0.011175
ENSSSCG00000028022	*COL6A2*	1.71	0.05198	1.36	0.029189
ENSSSCG00000026631	*SLC25A6*	1.73	2.74E-06	1.35	0.028352
ENSSSCG00000027060	*TBX2*	1.73	0.004374	1.39	0.026113
ENSSSCG00000026084	*MFSD7*	1.74	0.000551	1.41	0.019689
ENSSSCG00000012953	*YIF1A*	1.77	6.74E-08	1.33	0.038407
ENSSSCG00000000033	*BZRP*	1.82	0.03506	1.45	0.008436
ENSSSCG00000013901	*IFI30*	1.82	0.025505	1.37	0.035551
ENSSSCG00000022246	*ICOSLG*	1.83	0.001045	1.39	0.017888
ENSSSCG00000013622	*TMEM205*	1.83	2.45E-05	1.38	0.022633
ENSSSCG00000009668	*CLU*	1.92	0.000132	1.45	0.008532
ENSSSCG00000008508	*FAM98A*	1.95	2.27E-07	1.22	0.042541
ENSSSCG00000000675	*C1R*	1.99	3.56E-06	1.39	0.004673
ENSSSCG00000022236	*protein_coding*	2.01	0.008151	1.53	0.003243
ENSSSCG00000000606	*MGP*	2.04	0.008139	1.30	0.029075
ENSSSCG00000013074	*RAB3IL1*	2.16	5.39E-05	1.59	0.001274
ENSSSCG00000027466	*PCOLCE*	2.23	0.001512	1.27	0.033010
ENSSSCG00000013181	*SERPING1*	2.38	6.82E-07	1.27	0.029674
ENSSSCG00000012841	*ATGL*	2.39	6.54E-08	1.40	0.013320
ENSSSCG00000006276	*CEBPD*	2.57	3.62E-10	1.38	0.023386
ENSSSCG00000014834	*UCP3*	2.73	3.76E-09	1.68	0.000316

Hp – Hampshire; Pi – Pietrain; FC – fold change obtained for DEGs between different phenotypic groups in each breed
